# Testing for Differentially-Expressed MicroRNAs with Errors-in-Variables Nonparametric Regression

**DOI:** 10.1371/journal.pone.0037537

**Published:** 2012-05-24

**Authors:** Bin Wang, Shu-Guang Zhang, Xiao-Feng Wang, Ming Tan, Yaguang Xi

**Affiliations:** 1 Mathematics and Statistics Department, University of South Alabama, Mobile, Alabama, United States of America; 2 Department of Statistics and Finance, University of Science and Technology of China, Hefei, Anhui, People's Republic of China; 3 Department of Quantitative Health Sciences, Cleveland Clinic Foundation, Cleveland, Ohio, United States of America; 4 Mitchell Cancer Institute, University of South Alabama, Mobile, Alabama, United States of America; University of Turin, Italy

## Abstract

MicroRNA is a set of small RNA molecules mediating gene expression at post-transcriptional/translational levels. Most of well-established high throughput discovery platforms, such as microarray, real time quantitative PCR, and sequencing, have been adapted to study microRNA in various human diseases. The total number of microRNAs in humans is approximately 1,800, which challenges some analytical methodologies requiring a large number of entries. Unlike messenger RNA, the majority of microRNA (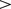
60%) maintains relatively low abundance in the cells. When analyzed using microarray, the signals of these low-expressed microRNAs are influenced by other non-specific signals including the background noise. It is crucial to distinguish the true microRNA signals from measurement errors in microRNA array data analysis. In this study, we propose a novel measurement error model-based normalization method and differentially-expressed microRNA detection method for microRNA profiling data acquired from locked nucleic acids (LNA) microRNA array. Compared with some existing methods, the proposed method significantly improves the detection among low-expressed microRNAs when assessed by quantitative real-time PCR assay.

## Introduction

MicroRNA (miRNA) is a panel of naturally-occurring non-coding RNA molecules in short length (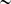
22 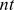 on average). To date, a total of 18,226 miRNAs, including 1,523 human miRNAs, has been registered in the miRbase database (Version 18.0). Evolutionarily conserved miRNA is capable of mediating approximately 30% of human genes, and involves many biological processes such as development, cell growth, differentiation, apoptosis, and tumorigenesis through its superior regulatory capability [1].

As a well-established discovery tool for biological and medical research, microarray technology has been migrated to the application of characterizing miRNA. Normalization is an essential matter for discovery experiments using microarray. It can minimize the systematic non-biological variations, and thus improves the identification of differentially-expressed miRNAs. The total number of miRNA is much smaller than messenger RNA (mRNA), which challenges the normalization methods utilizing global profiling information and requiring a large number of entries. Some methods including the cyclic loess method (LOESS) [2], [3], the modified-LOESS (LOESS-M) [4], and quantile normalization (QN) [5]–[7] have been applied to miRNA array analysis; however, the unique signature of miRNA, such as the small total number, has reduced the enthusiasm of direct adoption [8].

Measurement errors are introduced in miRNA microarrays from different sources, including sample preparation, dying, microarray hybridization, scanning, image intensity, and equipment errors, among many others. When the majority of miRNAs is weakly expressed, measurement errors dramatically increase the uncertainty in detecting the differentially-expressed miRNAs. In this study, we adopt and generalize the two-component measurement error model for miRNA microarray data, and propose to calibrate the measurement errors using an errors-in-variables nonparametric regression method (EIVNPR hereafter). Simultaneous confidence bands are constructed to test differentially-expressed miRNAs. The proposed methods are applied to LNA miRNA microarray profiling data accompanied with validation by qRT-PCR. The performances of the algorithms are evaluated by computing the weighted kappa statistic, which reveals the reproducibility between the two profiling methods, LNA array and qRT-PCR. Results show that EIVNPR efficiently calibrates the measurement errors and achieves better performance than the existing methods being benchmarked.

## Results

### Signal quality of the LNA miRNA profiling data

In miRNA data analysis, it is crucial to assess the signal quality of various profiles before normalization and differentially-expressed miRNAs detection. Signal-to-noise ratio (SNR) is a measure that compares the level of a desired signal to the level of background noise. MiRCURY LNA miRNA Array tests a set of 560 miRNAs with four technical replicates on each slide for each miRNA. In raw data processing, one signal intensity measure and one background intensity measure are obtained for each probe, and the negative and empty signals are flagged by the ImaGene 7.0 software. The outliers and poor signals, if the signal intensity is less than two standard deviations from the background intensity, are flagged automatically by the image processing software as well. We compute the SNR of a probe by dividing its net intensity (the background-subtracted signal) by the background. A ratio higher than 1/1 indicates that the signal is larger than the noise. For arrays with high quality signals, the SNRs tend to be large. To summarize the overall signal quality of a profile, a mean SNR is computed by taking the arithmetic average of the SNRs of all probes on a slide.

**Figure 1:**
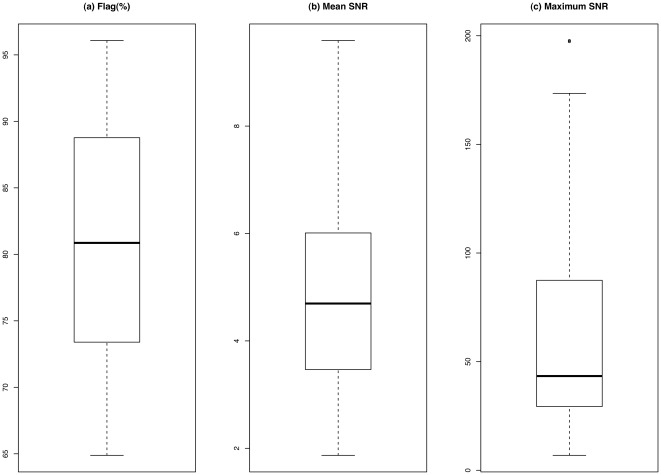
Signal quality evaluation for the LNA arrays. Plot (a) shows the boxplot of the percentages of flagged probes for all 40 profiles. Plots (b) and (c) show the boxplots of the mean and maximum signal-to-noise ratios, respectively.

The results in Figure 1 illustrate that the majority of human miRNAs are weakly or not expressed. Among all 40 human osteosarcoma xenografts profiles, at least 65% of the probes are flagged, and at least half of the profiles have more than 80% of the probes flagged (see panel (a)). All 40 profiles have mean SNR smaller than 10, and more than half of the profiles have mean SNRs smaller than 5.00 (see the panel (b) in Figure 1). Among all 40 profiles, the majority have maximum SNR smaller than 100, and five of which have maximum SNR less than 20 (see the panel (c) in Figure 1).

### Intra- and inter-platform reproducibility

The specimens are also evaluated using qRT-PCR. For each specimen treated with a specific chemotherapeutic treatment, a set of 663 miRNAs are tested with TaqMan Array (TLDA), with an overlap of 508 miRNAs tested with both TLDA and LNA miRNA Array. For each of the 508 miRNAs, the relative abundance is measured by the relative quantity (RQ) in qRT-PCR, and a fold-change given by the ratio between the mean intensity in the treated sample and the mean intensity in the control based on the LNA miRNA profiling data. For each of the two array platforms, TLDA and LNA array, the intra-platform reproducibility is assessed using the Spearman's correlation coefficient. That is, under each of the three chemotherapeutic treatments, a Spearman's correlation coefficient is computed between the two profiles of every pair of specimens from the same platform. To assess the inter-platform reproducibility, a Spearman's correlation coefficient is computed between the two profiles from TLDA and LNA array for the same specimen under the same chemotherapeutic treatment, respectively.

**Figure 2:**
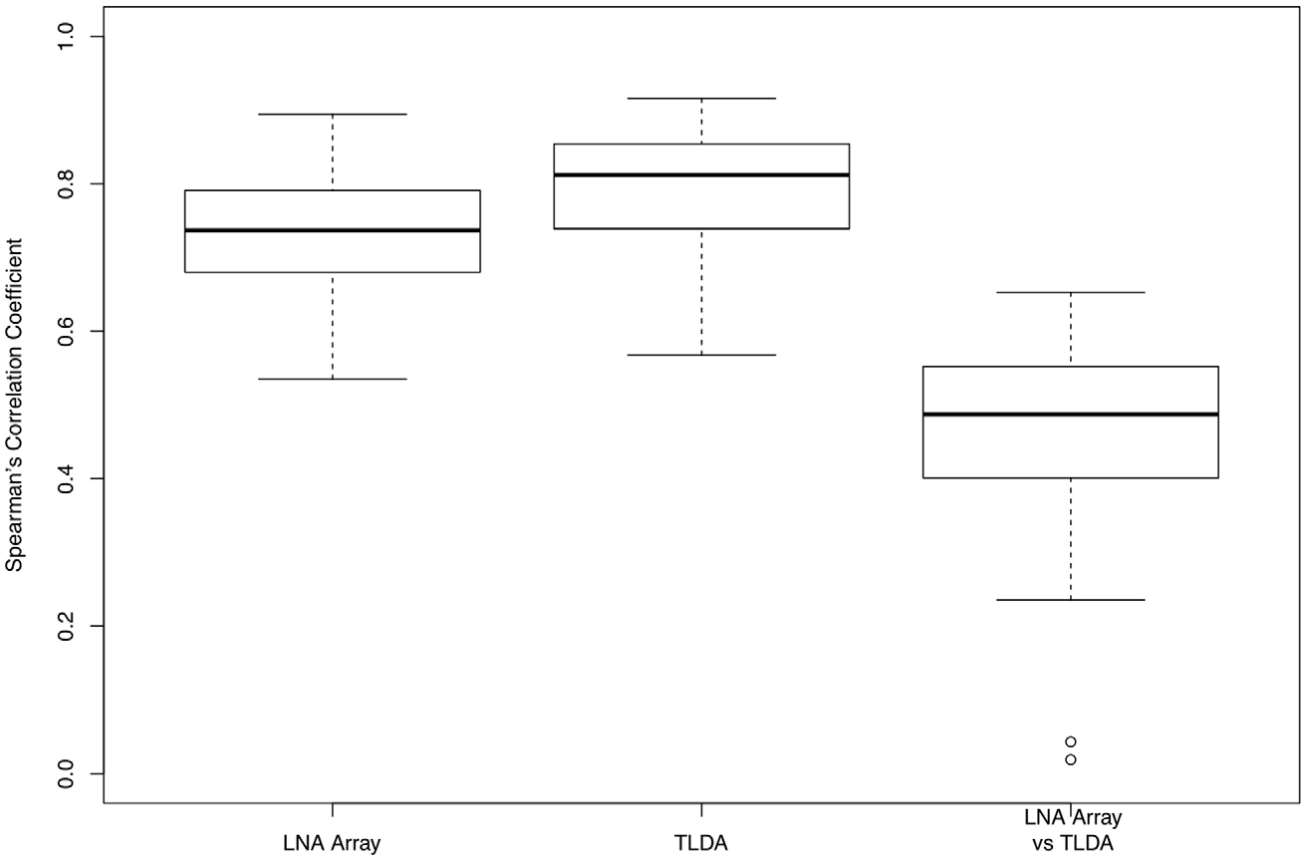
Intra- and inter-platform reproducibility for TLDA and LNA miRNA microarray. The panels to the left shows the boxplot of the Spearman's correlation coefficients between any pair of the 40 profiles obtained from LNA array; The boxplot in the middle shows the results for the qRT-PCR profiles. The panel to the right shows the boxplot of Spearman's correlation coefficients between the two profiles for the same sample obtained from LNA and TLDA arrays.

The left panel in Figure 2 shows the boxplot of the Spearman's correlation coefficients for all 30 profiles from LNA array, and the middle panel shows the qRT-PCR results from TLDA. We find that intra-platform reproducibility is high for both profiling methods. As revealed in the right panel, most of samples show high coefficients except sample 6. The inter-platform reproducibility is relatively low compared with the intra-platform reproducibility.

### Differentially-expressed miRNA detection without replicate arrays

For each specimen under a specific chemotherapeutic treatment, we obtained two profiles: one from the treated sample and one from the control. Due to the fact that the majority miRNAs is weakly expressed, we are facing a dilemma of whether or not to use the probe level measures that are weak. If we filter out the flagged probes, we may have too little available information to evaluate the regulation trends of most miRNAs, and we could dramatically over-estimate their expression levels by dropping the measures not significantly higher than the background noise. Another drawback is that some statistical tests such as the t-test can not be applied to detect the differentially-expressed miRNAs. On the contrary, if we keep measures from all probes, the test could be dominated by the measurement errors.

In this study, we filter out all probes from contaminated regions that are marked as outliers. Then detection of differentially-expressed miRNAs are performed based on (a) probes that are not flagged as weakly expressed, or (b) the rest of the probes including those are weakly expressed. When the flagged probes are filtered out, the number of usable probes are different for different miRNAs on each slide. We compute the mean intensity for each miRNA, and various normalization methods are applied to the two profiles of miRNAs with valid measures. As a result, some existing methods such as the t-test is not applicable for differentially-expressed miRNA detection. As a “poor-man's method”, regulation trends are identified using the fold-change (FC), which is the ratio between the intensity measures in the treated and control samples, or the difference of the logarithms of the intensity measures in the two samples, after normalization. Various FC cutoffs are used for the performance comparisons. Similar results are produced when a cutoff is selected between 1.5 to 2.2. A cutoff of two folds is adopted in the results reported in this study. For each normalization method, a classification table in the format of Table 1 is constructed for each treated sample, and a weighted kappa coefficient is computed to assess the reproducibility (or the degree of agreement) between the TLDA and LNA arrays. When all probes with weak signals are kept in the analysis, the differentially-expressed miRNAs can be detected using a t-test based on the technical replicates on each sides. If there are not enough usable probes, the FC method is used instead. Whichever method is used to detect the differentially-expressed miRNAs, the basel levels of the miRNAs are checked in identifying the regulation trends.

**Table 1:** Three-way classification table.

		*LNA results*			
		D.R.	ND.E.	U.R.	Total
	D.R.	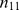	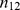	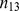	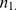
qRT-PCR	ND.E.	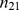	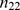	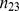	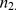
results	U.R.	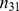	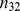	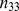	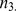
	Total	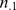	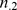	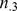	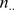

D.R. (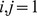) refers to down-regulated, ND.E. (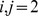)refers to non-differentially expressed, and U.D. (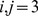) is for up-regulated. In the table 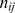 is the frequency of miRNAs. For instance, 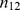 refers to the number of miRNAs that are classified to be down-regulated based on the qRT-PCR results, while classified to be non-differentially expressed based on the LNA results. The total and subtotals are defined as follows: 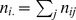, 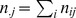, and 
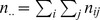
.

Results based on all 30 treated samples show that filtering out all flagged probes does not improves the reproducibility between the LNA array and TLDA results. The VSN and “invariants” methods won't work properly on the filtered data. The QN, LOESS-M and global median normalization methods produce similar results on filtered and unfiltered data. The EIVNPR method outperforms the existing normalization methods being benchmarked (see Table 2). By ignoring the probes flagged by the image processing software, EIVNPR results in slight agreement for 21 samples and no agreement for the other samples (see column marked EIVNPR2 in Table 2 and Table 3). If we keep the measures of all probes, after normalization with EIVNPR, 18 samples show slight agreement, four samples show fair agreement and one sample show moderate agreement (detailed results in column marked as EIVNPR1 in Table 2).

**Table 2:** Weighted kappa coefficients computed based on all probes.

	EIV-	EIV-					
Sample	NPR1	NPR2	VSN	Inv	L-M	QN	Med
CIS-1	0.09	0.05	0.04	0.05	0.01	−0.06	0
DOX-1	−0.03	−0.13	−0.09	−0.2	0	−0.19	−0.07
IFO-1	0.2	0.07	−0.08	0.07	0	0.14	0.29
CIS-2	0.41	0.04	−0.09	−0.01	−0.11	−0.22	0.04
DOX-2	0.08	0.07	−0.07	−0.01	0	0.04	0
IFO-2	0	0.12	0.05	−0.07	0.06	0.12	−0.05
CIS-3	0.07	0.04	0.01	0.02	−0.03	−0.12	0.03
DOX-3	0.28	0.06	−0.03	0.09	0.4	0	0
IFO-3	0.02	0.14	0.05	−0.07	0.01	0.03	0.09
CIS-4	0.1	0.03	0.04	0.03	0	0.02	0.09
DOX-4	0.06	−0.14	−0.04	−0.01	0.01	−0.19	−0.01
IFO-4	0.1	0.09	0.02	0.04	−0.21	0.07	0.05
CIS-5	−0.1	−0.13	−0.02	0.06	0	−0.02	0.03
DOX-5	−0.11	−0.07	0	−0.01	0	0.02	0.04
IFO-5	−0.03	0	0.05	0.04	0	−0.05	−0.04
CIS-6	−0.1	−0.02	−0.03	−0.03	0	0.03	−0.1
DOX-6	−0.02	−0.02	−0.01	0.02	0	0	−0.05
IFO-6	0.06	0.01	−0.1	−0.2	0	−0.05	0
CIS-7	0.04	0.08	0	0.14	0	0.04	0.06
DOX-7	0.02	−0.03	0	−0.03	0	0.04	−0.02
IFO-7	0.19	0.17	0	0.02	0	0.03	−0.01
CIS-8	−0.1	0.05	0.06	−0.05	0	0.01	−0.11
DOX-8	0.23	0.17	0	0.05	−0.03	0.01	0.01
IFO-8	0.19	0.11	0.04	0.05	−0.06	−0.02	0.07
CIS-9	0.16	−0.03	0.02	0.1	0.16	−0.05	−0.01
DOX-9	0.03	0.13	0	0.06	0	−0.03	0.03
IFO-9	0.04	0.04	−0.04	0.04	−0.01	−0.09	0.19
CIS-10	0.16	0.14	-0.01	0	0	0	0.01
DOX-10	0.11	0.13	−0.02	0.05	0	0	0.15
IFO-10	0.2	−0.01	0.01	0.01	0	0.04	0.02

The first two columns show the specimen IDs and the names of the chemotherapeutic treatments: Cisplatin (Cis), Doxorubicin (Dox), and Ifosfamide (Ifo). The results for the following six normalization methods are shown in columns 3 through 8, respectively: “EIVNPR1” based on all probes, “EIVNPR2” with flagged probes excluded, “VSN”, “Inv” for normalization by “invariant”, “L-M” for LOESS-M, “QN” for quantile normalization, and “Med” for global median normalization.

**Table 3:** Normalization comparisons based on weighted kappa test.

	Agreement					
					Sub-	Almost
Method	No	Slight	Fair	Moderate	stantial	Perfect
EIVNPR	7	18(7)	4	1	0	0
EIVNPR2	9	21(8)	0	0	0	0
VSN	13	17(0)	0	0	0	0
Invariants	11	19(2)	0	0	0	0
LOESS-M	5	23(1)	0	1	0	0
QN	12	17(2)	0	0	0	0
Median	10	19(2)	1	0	0	0

Normalized with the other existing normalization methods without filtering out the flagged probes, the majority of the 30 samples show slight agreement, according to Landis and Koch's interpretation of the Kappa test statistic. To further compare the performances of various normalization methods, we checked the samples having weight kappa coefficients greater than 0.1 (the number of samples are shown in parentheses in column 3 of Table 3). We find that VSN results in 30 weight kappa coefficients that are smaller than 0.1, while this number is 29 for LOESS-M, 28 for “invariant” method, median normalization and quantile normalization (QN), respectively. In addition, LOESS-M results in one sample show moderate agreement, and median normalization results in one sample show fair agreement. It is worth noting that LOESS-M results in less no agreement than the other methods, and its performance is close to EIVNPR based on the filtered profiles.

### Performance comparisons with replicate measures from multiple profiles

We further pool the arrays for all 10 specimens together under the same chemotherapeutic treatment as biological replicates. Hence, we have 10 replicate arrays from the treated samples for each treatment, and for 10 controls. To apply EIVNPR, we first normalize multiple arrays using the built-in normalizers. Twelve normalizers are provided on each LNA miRNA array for normalization purposes. They are hsa_SNORD2, hsa_SNORD3, hsa_SNORD4A, hsa_SNORD6, hsa_SNORD10, hsa_SNORD12, hsa_SNORD13, hsa_SNORD14B, hsa_SNORD15A, hsa_SNORD118, U6-snRNA-1, and U6-snRNA-2. These normalizers are supposed to highly and stably express across experiments. We first filter these normalizers using the flag information by the ImaGene 7.0 software. Second, we compute the normalization parameter for each profile using a maximum likelihood based iterative algorithm as in [9]. In the iterative algorithm, each normalizer is tested and will be removed if it has a significantly larger dispersion than the others. In case there are not enough normalizers, we expand the search to include the spike-ins on each array. Third, we compute the average log-transformed intensities and standard errors for all miRNAs under the treatment and control, respectively. Last, we apply EIVNPR to detect the differentially-expressed miRNAs using the 95% confidence bands.

Based on the 10 profiles from the treated samples and the 10 profiles as controls, we applied normalization methods such as QN, LOESS, LOESS-M, and median normalization, respectively. A paired t-test is performed to detect the differentially-expressed miRNAs based on multiple normalized profiles. The first column in Table 4 shows the names of the miRNAs classified as differentially-expressed by various methods, based on the specimens under treatment Ifo. The second column gives the qRT-PCR results: a t-test is applied based on the logarithms of the 10 RQ values to test whether the true RQ value is significantly different from zero (or equivalently the miRNA is differentially-expressed). From Table 4 we find that by applying LOESS normalization and t-test, only hsa-miR-27 is identified as differentially-expressed, which is validated by qRT-PCR results. If the LOESS-M normalization method is applied instead, hsa-miR-24 will be identified as differentially-expressed as well, which is also validated by qRT-PCR results. With QN, hsa-miR-22 and hsa-miR-143 are detected and validated by qRT-PCR results, but hsa-miR-30e is misclassified at significance level 0.05. The median normalization detected four miRNAs and all are validated by qRT-PCR results, but unfortunately none is consistent with those by QN, LOESS, and LOESS-M. If we simply normalize the profiles using the normalizers, and detect the differentially-expressed miRNAs by a t-test, hsa-miR-191, hsa-miRlet-7b, hsa-miR-24 and hsa-miR-130b are correctly detected (column 4 marked as “ME”). Using the 95% confidence bands method from EIVNPR, a total of 10 miRNAs are detected with seven validated by qRT-PCR, and the other three are misclassified. Among the seven miRNAs validated by qRT-PCR, two miRNAs have p-value 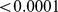 with one identified by median normalization, and four mIRNAs have 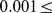
p-value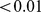 where hsa-miR-27a is also detected by LOESS and LOESS-M.

**Table 4:** Comparisons of differentially-expressed miRNA detection (I).

hsa-miR-	RQ	Med	ME	QN	L	L-M	EIVNPR
142-3p	***						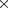
191	***		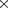				
199a-3p/199b-3p	***	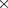					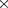
let-7b	**		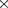				
27a	**				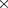	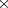	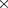
27b	**	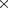					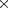
103	**						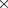
584	**						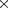
22	*			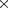			
24	*		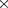			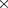	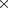
30b	*	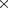					
130b	*		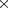				
143	*			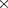			
503	*	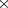					
30e	.			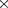			
19a							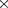
19b							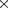
623							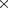

(1) “Med” = median normalization; “ME” = normalized using normalizers; “L” = LOESS normalization; “L-M” = LOESS-M normalization. (2) Symbols in column 2: “***” if p-value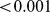; “**” if 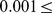
p-value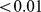; “*” if 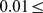
p-value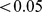; “.” if 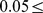
p-value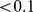; none otherwise.

Table 5 shows the detailed classification results by different methods. The first column shows a sequence of classes for the p-values from 0 to 0.05. For each method listed in the first row, a p-value is computed for each miRNA. The number of miRNAs with p-values fall in a specific class is shown as 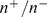, where 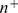 is number of miRNAs that are correctly classified as differentially-expressed (TP: true positive), and 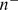 is the number of miRNAs that are incorrectly classified as differentially-expressed (FP: false positive). From Table 5, we see that if we lower the significance level from 0.05 to 0.03, EIVNPR can correctly identify five miRNAs with no FPs. The median normalization can detect one less miRNAs than EIVNPR at significance level 0.03 with no FP as well. If we further lower the significance level to 0.025, we see all methods will produce similar results, except that QN has one FP and LOESS can detect only one differentially-expressed miRNA.

**Table 5:** Comparisons of differentially-expressed miRNA detection (II).

p-value	Med	ME	QN	L	L-M	EIVNPR
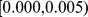					2/0	1/0
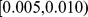			1/0			
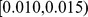			0/1	1/0		1/0
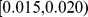	2/0	1/0	1/0			
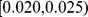		1/0				
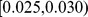	2/0					3/0
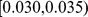						0/2
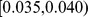						1/0
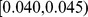						
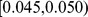		2/0		1/0		1/1

The classification results are shown as 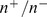, where 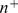 is the number of miRNAs that are correctly classified as differentially-expressed (true positive), while 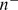 is the number of miRNAs that are incorrectly classified as differentially-expressed (false positive).

## Discussion

The “invariants” method is developed specifically for miRNA analysis by finding a set of stably and highly expressed miRNAs for normalization [10]. However, it is challenging to find such a set of “invariants”, especially when the expression levels of the majority of miRNA are close to the background noise due to their relatively low abundance in the cells. For miRNA profiling data, normalization methods based on the designed “normalizers” is feasible and reliable. However, it is also found that some endogenous normalizers are not stable across experiments [11]–[13].

The relationship between two gene expression profiles is usually non-linear, especially for the genes/miRNAs with extremely high expression levels. In concern of the nonlinearity, several prevalent nonlinear normalization methods such as LOESS, LOESS-M and QN, are adapted for miRNA study with or without modification. LOESS is a method based on the idea of the M-A plot by regressing 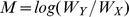 on 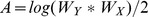 via locally weighted polynomial regression. The LOESS-M is a modification of LOESS by subtracting the median of 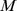 from the loess fit to the MA-plot. QN assumes that various profiles have a common distribution, and all profiles are forced to have the same quantiles at all levels. The common distribution assumption is reasonable for mRNA or cDNA data normalization because most genes are strongly and non-differentially expressed, and the total number of entries is very large. However, these assumptions might not hold true for miRNA data. VSN is another popular microarray data normalization method via a variance stabilization transformation to expression data [14]. Literature shows that both the invariant and quantile method achieved satisfying performances for one-color miRNA microarrays [10], [15].

The errors-in-variables nonparametric regression method can effectively calibrate the measurement errors and improve the detection of differentially-expressed miRNAs. It can be applied to multiple profiles normalized by some existing popular normalization methods, or by a measurement error model-based normalization procedure as in [9]. When EIVNPR is applied to two profiles, one treatment and one control, the normalization step can be bypassed for the purpose of differentially-expressed miRNA detection. On the other hand, EIVNPR is computational intensive and sensitive to the following issues. First, the results are sensitive to bandwidth selection. If one prefers to have a more smooth regression curve, the regression model is supposed to be more robust to outliers. A trade-off is that the result for an individual miRNA might be affected too much by the other miRNAs having similar expression levels. If the bandwidth is too small, the fitted curve will become too bumpy. The data-driven adaptive bandwidth selector proposed in this study works pretty well. Second, when no or less replicates are available, finding good estimates of the variances of the measurement errors is challenging. When we have only one treatment and one control, we can estimate the variances using the measures in a close neighborhood.

**Figure 3:**
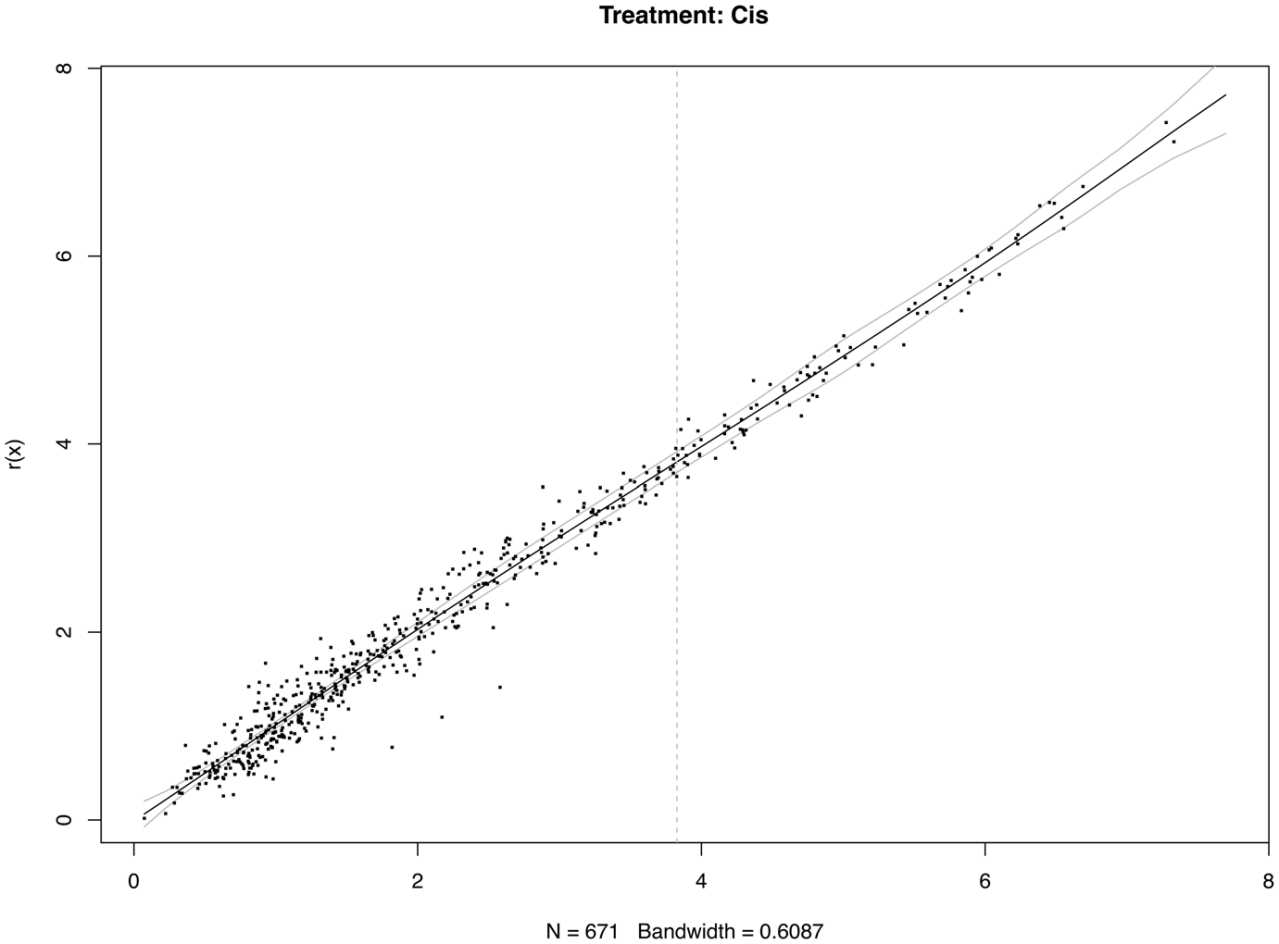
Differentially-expressed miRNA detection using simultaneous confidence bands (under treatment Cis, with replicated arrays).

**Figure 4:**
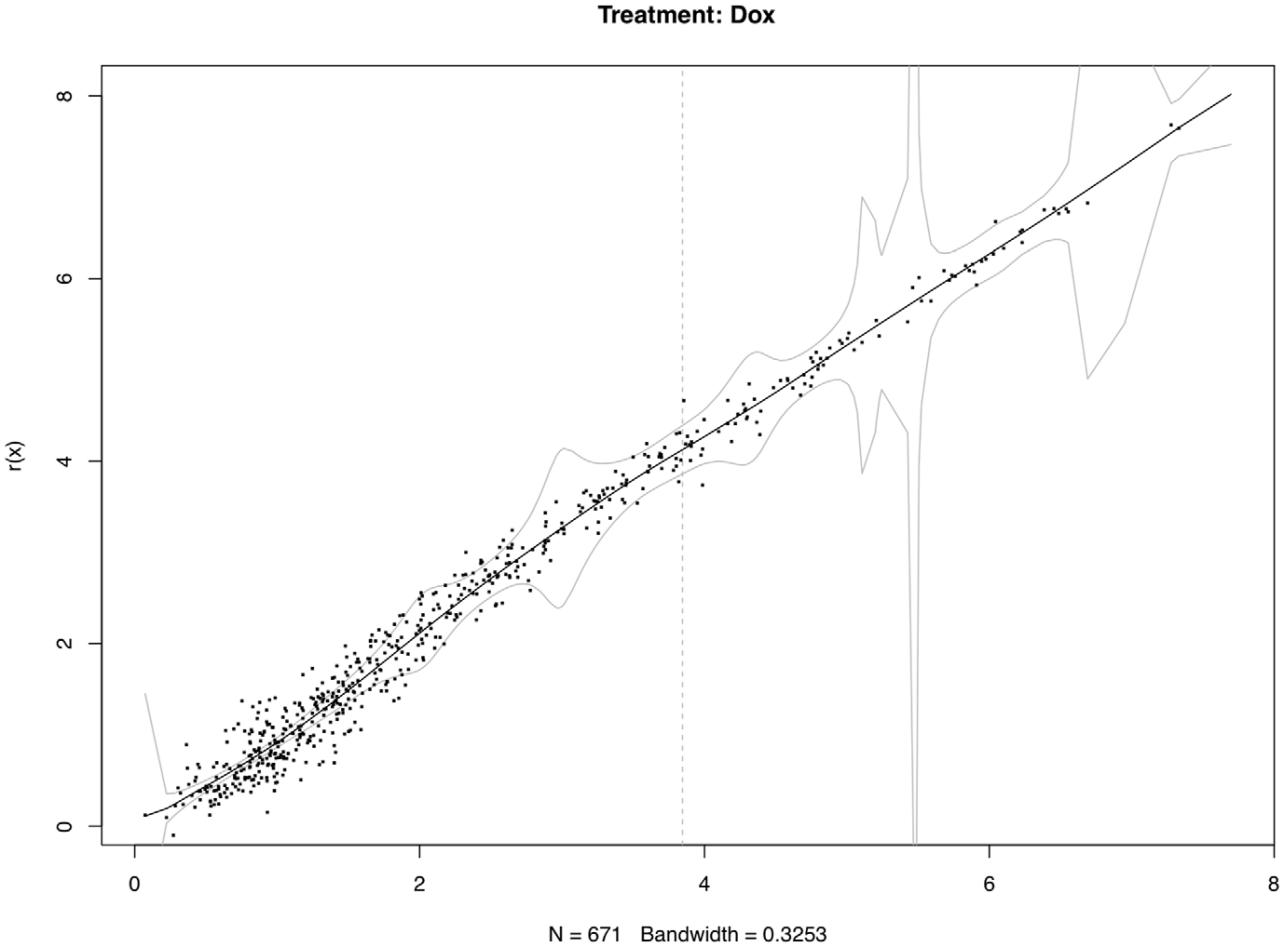
Differentially-expressed miRNA detection using simultaneous confidence bands (under treatment Dox, with replicated arrays).

**Figure 5:**
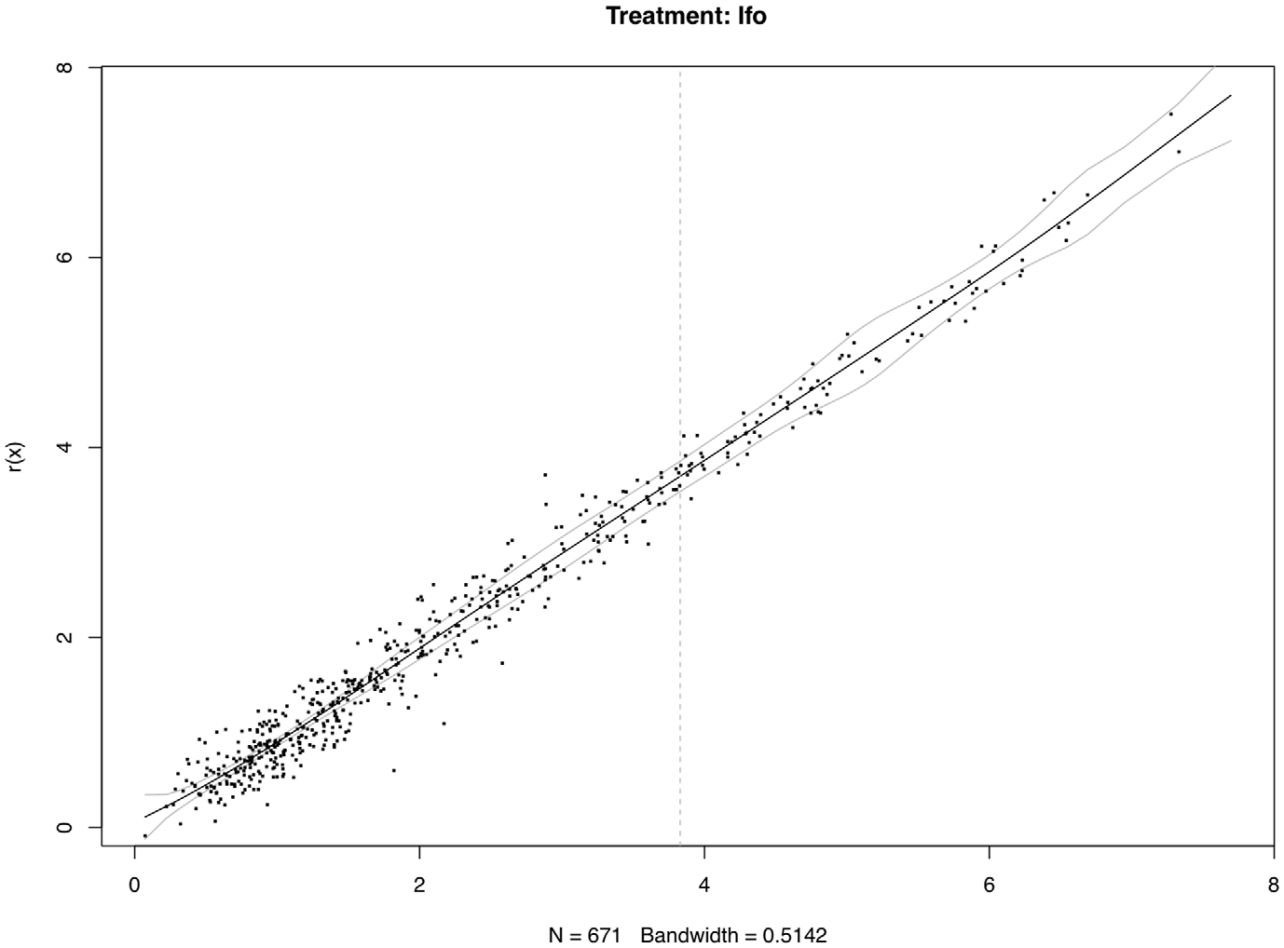
Differentially-expressed miRNA detection using simultaneous confidence bands (under treatment Ifo, with replicated arrays).

**Figure 6:**
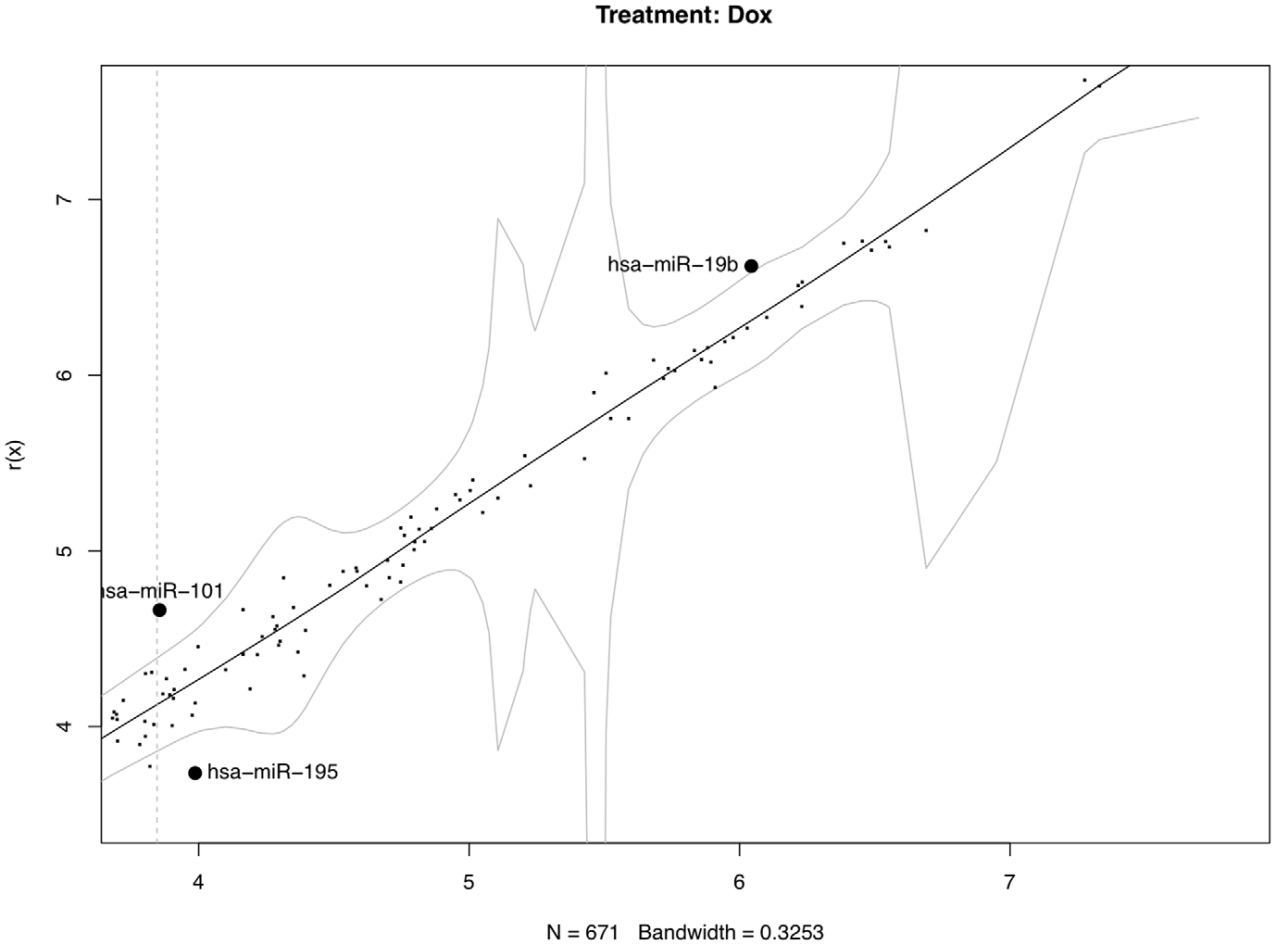
Differentially-expressed miRNA detection using simultaneous confidence bands (under treatment Dox, with replicated arrays).

It is worth noting that the proposed method might be a little bit aggressive. Using simultaneous confidence bands based on non-parametric regression can utilize more global information to a large extent. However, it ultimately increases the risk of misclassification as a trade-off. There is no clear cutoff as for whether a signal is strong or weak; classifications of the differentially-expressed miRNAs should be done by using both the simultaneous confidence bands, and basal levels of the individual miRNAs. Figures 3, 4, 5 show the 95% confidence bands based on the specimens under the three treatments, respectively. We see that the regression curve is pretty smooth in all three figures. But the confidence bands in Figure 4 is not very smooth. In each of these three figures, a vertical line is drawn to mark the position two standard deviations above the mean background noise (log-transformed). When we detect the differentially-expressed miRNAs, this line can be used as a reference to check the strength of the signals. From Figure 6, we see three miRNAs fall outside the 95% simultaneous confidence bands: hsa-miR-19b has strong signal, but it stays very close to the upper band. The other two miRNAs, hsa-miR-101 and hsa-miR-195, stay farther from the confidence bands, but their expression levels are not very high. The qRT-PCR results show that none of these three miRNAs are significantly-expressed.

### Conclusions

Data quality assurance is crucial in miRNA array data analysis. Well designed and well performed experiments can alleviate the bias from various sources, but can not completely eliminate the measurement errors. For miRNA microarray data, the signal quality is not as good as that for mRNA/cDNA microarray data. The majority of miRNAs are often weakly or not expressed, and the rest may have overall low SNR, which increases the uncertainty in detecting the differentially-expressed miRNAs. By modeling the measurement errors with a two-component measurement error model, and calibrating the measurement errors with errors-in-variables nonparametric regression, the proposed method using simultaneous confidence bands is more sensitive to detect the differentially-expressed miRNAs. At the same significance level, the proposed method tends to classify more miRNAs are differentially-expressed than the other existing methods, and increases the false positive rate as a trade-off. However, as a conservative solution we can lower the significance level to achieve similar false positive rates (see Table 5). Potentially the proposed method can improve the inter-platform reproducibility and can be applied for cross-platform and/or cross-lab microarray data integration.

## Materials and Methods

### Sample preparation and profiling data acquisition

Each of ten specimens are treated with three chemotherapeutic treatments: cisplatin(Cis), Doxorubincin (Dox), and Ifosfamide (Ifo), respectively. In addition, each specimen is treated with saline and is used as a control to detect the differentially-expressed miRNAs under different chemotherapeutic treatments. The 40 human osterosarcoma xenografts were prepared as previously described in [16]. RNA was isolated, purified, and quantified using established protocols [17]. The miRCURY LNA microRNA Array based on miRbase 9.2 (Exiqon Inc., Denmark) and TaqMan Low Density Array (TLDA) Human MicroRNA Panel v2.0 (Applied Biosystems, CA, USA) were employed for miRNA global profiling and data validation, respectively. The detailed procedures are referred to in our previous publication [12], and raw data are available at http://gauss.usouthal.edu/publ/ada/.

### Measurement error models for gene expression data

In gene expression arrays it is observed that the standard deviations of measurements are proportional to the expression levels; and this proportionality cannot continue down for entirely unexpressed genes – the standard deviations of the weakly or non-expressed genes won't be zero [18]. A two-component measurement error model, which was originally developed in the context of instrumental methods of analytical chemistry, was extended for gene expression arrays [19]–[21]. In the same spirit, we consider the following measurement error models for one-color miRNA microarrays:
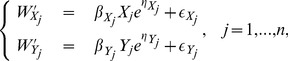
(1)where 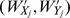 is a pair of net median fluorescent intensities (nMFI's), which is the background-subtracted response at concentration 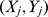, and 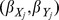 is a pair of relative expression levels that are usually indiscernible unless extra calibration data are available. In (1), two types of measurement errors are considered: 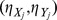 represents the multiplicative error that always exists but is noticeable at concentrations significantly above zero, and 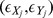 represents the additive error that always exists but is noticeable mainly for near-zero concentrations. In this study, we assume independence among the error terms with 
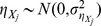
, 
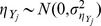
, 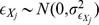, and 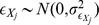. In addition, heteroscedastic errors are assumed for both the additive and multiplicative errors in the models in (1).

Applying a Taylor expansion to the logarithm of 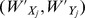, we get
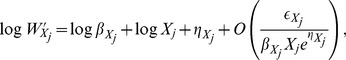
(2)

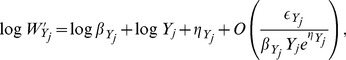
(3)where the higher order terms to the right-hand side of the above two equations are negligible when miRNA-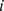 is not weakly expressed in the two cell populations. Let 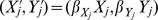. When the higher order terms are absorbed into the multiplicative errors in (2) and (3), we get
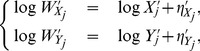
(4)where 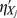 and 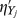 are independent heteroscedastic normal errors with mean zero and standard deviations 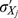 and 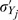, respectively.

### Statistical inference through nonparametric regression with errors-in-variables

In order to identify the differentially-expressed miRNAs, we propose a statistical inference approach through constructing simultaneous confidence bands (SCB) under an errors-in-variables regression model.

We are interested in the nonlinear relationship between the uncontaminated (log-transformed) intensities 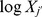 and 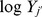. A conventional regression model can be formulated as
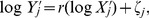
(5)where 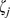 is the random error with 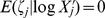. The regression function 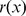 is the expectation of 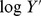 on the condition that 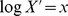, *i.e.*, 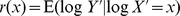.

Directly estimating 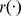 is not feasible since 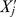 and 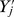 are the true expression levels of miRNA-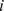 in the two cell populations and are unobservable. However, combining (4) and (5) results in an errors-in-variables regression model,
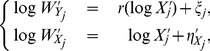
(6)where 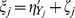 is the random residual error and 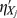 is the measurement error. Notice that 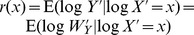 since 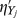 is independent of 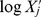 with mean zero. Therefore, 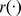 can be estimated from the observed contaminated data 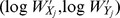 using the local polynomial deconvolution estimator [22]. In this study, both the random error 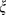 and the measurement error 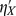 are heteroscedastic [23]. For the random error, we simply assume that it has a very general variance function, 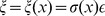, where 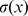 is the unknown variance function and 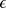 has mean 0 and variance 1. For the measurement error, the heteroscedastic variance parameters can be estimated directly from the data.

We consider a local linear deconvolution estimator; it is a special case of local polynomial estimator with degree 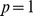. It is given by
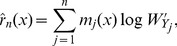
(7)where
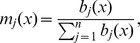


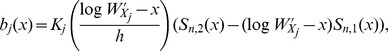


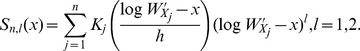
For heteroscedastic normal errors, we consider the following kernel for 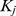,
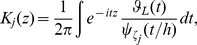
where
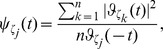


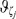
 is the characteristic functions of the 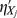, 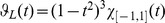 with the indicator function 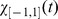, and 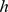 is the smooth parameter [24].

We then construct the 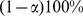 SCB for 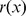 to identify the differentially expressed miRNAs. The observations that do not fall into the confidence regions are considered as the differentially expressed miRNAs. One advantage of the approach is that the normalization step is by-passed in identifying the differentially expressed miRNAs. The form of the 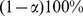 confidence bands for 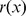 over a subset 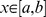 of the predictor space is taken by
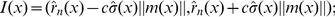
(8)for some 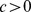, where 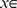 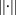 denotes the 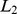 norm. To obtain 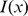 in (8), we need to calculate the critical value 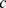 and the residual variance function 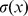. 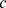 can be found using the *tube formula* [25], [26],
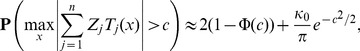
(9)where 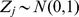 and 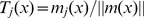, and
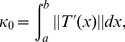
(10)where 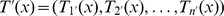 and 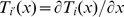.

There are two approaches to estimate 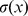. We may take the nonparametric estimator proposed by [27]. It is given by
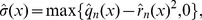
(11)where 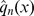 is the local linear estimate of 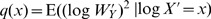. To avoid zero estimates in (11), one further implements the bagging-type correction algorithm to compute 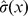 [27]. When the level of measurement errors are relatively small, we may use the other simple method by ignoring the measurement error effect on the variance function (See more discussions of the effects of error magnitude in measurement error models in [28]). The following procedure is adopted from [29]: first, define 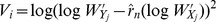; second, regress the 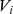
's on the 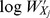
's using any nonparametric method to get an estimate 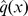 of 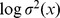 and compute 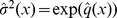.

For microarray data, the distribution of the intensities after logarithm transformation is usually still skewed. We use a variable bandwidth to choose the smoothing parameter 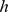 by following the idea of the conventional adaptive kernel estimator in [30]. First, find a pilot estimate 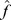 of the density function of 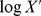 based on 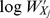, with bandwidth 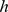 and with measurement error considered; second, define local bandwidth factor 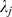 by taking 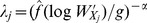, where 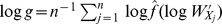, and 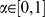 is the sensitivity parameter; finally, define a bandwidth 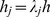. The smoothing parameter 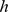 and the sensitivity parameter 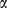 are selected by minimizing the *leave-one-out cross-validation score* 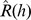 defined as
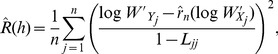
(12)where 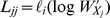.

Details of implementation of the errors-in-variables non-parametric regression and the construction of SCB are described in the R script available at http://gauss.usouthal.edu/publ/ada/.

### Normalization methods for benchmarking

All normalization methods are performed in R, an open source statistical scripting language (http://www.r-project.org). Median normalization is performed by dividing each array by its median signal intensity, and then by rescaling them to the global median intensity of all arrays. A function “normalize.quantile” from R package *affy* can be used to perform the quantile normalization [5]. The traditional LOESS normalization method is based on the idea of the 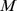 versus 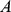 plot, which has been implemented in an R packages *codelink* and *affy* [2], [3]. The LOESS-M normalization is a modification of the traditional loess normalization by subtracting the median of 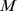 from the loess fit to the MA-plot 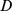 [4]. R functions were written to implement the LOESS-M normalization. Invariants normalization is performed based on a set of probes that have medium-high mean intensity and low variance across arrays (named “invariants”) [10]. R script at http://www.unil.ch/dafl/page58744.html is used. VSN normalization is performed using the “vsn2” function from R package “vsn” from the Bioconductor project (http://www.bioconductor.org).

### Weighted kappa test for platform reproducibility evaluation

Sensitivity and specificity are commonly used to evaluate the reproducibility or consistency between two platforms when interests are focused on whether the miRNAs (genes) being studied are differentially-expressed or not. In this study, instead of classifying the miRNAs as differentially-expressed and non-differentially expressed, we further identify the regulation trends. When the regulation trends are also of concern, sensitivity and specificity are not convenient to be used to compare the performances of different methods for a three-way classification [9], [11]. A three-way classification table is presented in Table 1. We adopt the weighed kappa test to measure the agreement between two qualitative classification schemes:
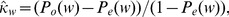
(13)where 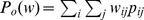, 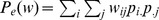, 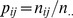, 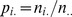, 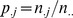. We define a distance 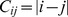 to quantify the relative difference between categories, and use the Fleiss-Cohen weighting scheme to compute 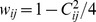 [11], [31]–[34]. The degree of agreement can be interpreted as follows: no agreement if 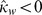, slight agreement if 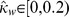, fair agreement if 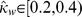, moderate agreement if 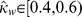, substantial agreement if 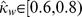, and almost perfect agreement if 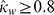 [35].
